# Cancer Incidence Among Air Transportation Industry Workers Using the National Cohort Study of Korea

**DOI:** 10.3390/ijerph16162906

**Published:** 2019-08-14

**Authors:** Wanhyung Lee, Mo-Yeol Kang, Jin-Ha Yoon

**Affiliations:** 1 Department of Occupational and Environmental Medicine, Gil Medical Center, Gachon University College of Medicine, Incheon 21565, Korea; 2 Department of Occupational and Environmental Medicine, Seoul St. Mary’s Hospital, College of Medicine, The Catholic University of Korea, 222, Banpo-daero, Seocho-gu, Seoul 06591, Korea; 3 The Institute for Occupational Health, Yonsei University College of Medicine, Seoul 03722, Korea; 4 Department of Preventive Medicine, Yonsei University College of Medicine, Seoul 03722, Korea

**Keywords:** air transportation industry workers, neoplasms, leukemia

## Abstract

Background: There are increasing concerns regarding increased cancer risks in professional flight attendants due to their exposure to occupational hazards that are known or suspected to be carcinogenic. In this study, we aimed to analyze various cancer risks among a cohort of Korean air transportation industry workers. Methods: We used data from the Korean National Health Insurance Service (NHIS) database from 2002 to 2015. The age-standardized incidence ratios (SIRs) for all types of cancers in the aircraft transport industry workers compared to government employees and the entire employee population were calculated with adjustment for five-year age ranges via the indirect standardized method with gender stratification. Results: Leukemia (ICD-10; C91–C95) showed significantly higher SIRs (95% confidence interval (CI)) compared to the government employee group (1.86, 1.15–2.84) and the whole employee group (1.77, 1.10–2.70). Conclusion: Air transportation industry workers have an increased risk of leukemia compared to other occupational groups.

## 1. Introduction

As globalization continues and air travel becomes a more common mode of transportation, there are increasing concerns regarding increased cancer risks in professional flight attendants because of their exposure to occupational hazards known or suspected to be carcinogenic. Aircraft workers are exposed to various chemical and physical hazards, such as jet engine emissions, ionizing radiation, electromagnetic fields (EMFs) from cockpit instruments, ultraviolet radiation, circadian rhythm disruption, low atmospheric pressure, poor air quality, cigarette smoke, ozone, pesticides, and other volatile substances emanating from aircraft construction materials [1,2,3,4,5]. Several studies have reported these hazardous environments as risk factors for injury, musculoskeletal disorders, reproductive disorders, mental illness, infectious diseases, acquired immunodeficiency syndrome (AIDS), alcoholism, intentional self-harm, and even cancer [5].

The first reports on cancer incidence and mortality among civil and military aircraft crew were published in the early 1990s, mainly in Europe and North America [6,7,8]. These studies assessed the cancer incidence and mortality in aircraft crew and whether exposure to radiation of cosmic origin increased their risk of cancers and other diseases. Since then, several studies have been conducted regarding the development of cancers in flight crew. Previous studies have reported the increased incidence of specific cancers, predominantly skin cancer, breast cancer, prostate cancer [9,10], and brain cancer [11] in flight attendants. However, the incidence tended to be relatively small in these studies. Therefore, several meta-analyses have also been conducted [12,13,14]. A meta-analysis published in 2000 reported that flight personnel were at increased risk of several types of cancers, including carcinoma of the colon, prostate, and brain, and melanoma among men; and breast cancer, colon cancer, and melanoma in women [12]. Recently, a well-designed pooled analysis involving more than 93,000 aircraft crew members from 10 countries was conducted [15]. The standardized mortality ratios (SMRs) were calculated and the all-cause SMR (95% confidence interval (95% CI)), except for accidents, was 1.06 (0.98–1.15) for aircraft crew.

However, while the results of previous studies have been derived from mortality data, due to the high survival rates of many cancers, these results are less informative. Moreover, there are few areas outside Nordic countries with several decades of population-based registration of cancers. Hence, there remains controversy regarding the relationship between occupational exposure among air transportation industry workers and the risk of cancers. In this study, we aimed to analyze various cancer risks among a nationally representative cohort of Korean air transportation industry workers with the objective of providing scientific evidence regarding the risk of cancers in this population.

## 2. Methods

### 2.1. Data

We used data from the Korean National Health Insurance Service (NHIS) database from 2002 to 2015. By law, the National Health Insurance System of Korea covers most of the citizens residing within the territory of Korea (in 2015 approximately 98% of the 52,034,424 persons living in Korean territory were covered by the NHIS) [16]. The NHIS data include qualification data and claims information related to medical services. The qualification data in the NHIS database include patient age, sex, region, income, type of insurance, job and industry category, identification number, and family information. The medical service data include records of all covered inpatient and outpatient visits, procedures, and prescriptions for diseases categorized under the standardized protocol of the Korea Classification of Diseases and Causes of Death 4th edition, which corresponded to the International Classification of Diseases, 10th revision (ICD-10). All disease diagnoses are described via the ICD-10 codes.

### 2.2. Study Participants and Cohort

The study participants were selected from the employee subscribers from the qualification data in the NHIS who were aged 25–60 years between 2002 and 2015. The air transportation industry workers were categorized as transport workers in section H “transportation and storage” in the most recent Korean national standardized industrial classification developed by the Korea National Statistical Office following the fourth revision of the International Standard Industrial Classification of All Economic Activities (ISIC) in 2008 [17]. We then defined air transportation industry workers as those working in “Passenger air transport” service, such as charter flights for passengers, scenic and sightseeing flights, or general aviation activities, as well as those employed in “Freight air transport” service, such as transport freight by air over regular routes and on regular schedules, non-scheduled transport of freight by air, or renting of air-transport equipment with operators for the purpose of freight transportation excluding “Warehousing and support activities for transportation”.

The group of interest was defined as workers who continuously worked in the air transportation industry for the first three years (2006–2008). The government employee group was identified based on the type of insurance determined from the qualification data of the NHIS, including public officers or private school staff who were covered by NHIS under the government employee pension service. The whole employee cohort was defined as workers who were eligible employee subscribers based on the qualification data from the NHIS. The person-years of both reference groups were estimated from the “enroll year” to the “retired year” based on the changing qualification types in the NHIS database.

### 2.3. Cancers

The NHIS claims for inpatient and outpatient visits, procedures, and prescriptions were coded using the ICD-10, which was adopted in Korea in 1995, as well as the Korean Drug and Anatomical Therapeutic Chemical Codes [18]. Cancers were defined for inpatients with claims information with the ICD-10 code “C00-C97 Malignant neoplasms” as the main disease.

Malignant neoplasms were classified into seven groups and 27 subgroups based on the Korean Standard Classification of Diseases (KCD) from the ICD-10 code according to the human organ systems [19,20]. The gastrointestinal tract system group included malignant neoplasms of the lip, oral cavity, and pharynx (C00–C14), esophagus (C15), stomach (C16), colon (C18), rectosigmoid junction, rectum, anus, and anal canal (C19–C21), liver and intrahepatic bile ducts (C22), pancreas (C25), and other digestive organs (C17, C23, C24, and C26). The respiratory system group included malignant neoplasms of the larynx (C32), trachea, bronchus, and lung (C33, C34), and other respiratory and intrathoracic organs (C30, C31, and C37–C39). The bone and skin group included malignant neoplasms of the bone and articular cartilage (C40 and C41), malignant melanoma of the skin (C43), other malignant neoplasms of the skin (C44), and malignant neoplasms of the mesothelial and soft tissue (C45–C49). The genitourinary tract system groups were defined differently by sex. The male genitourinary tract system group included malignant neoplasms of the prostate (C61), other male genital organs (C60, C62–63), bladder (C67), and other malignant neoplasms of the urinary tract (C64–C66, C68). The female genitourinary tract system group included malignant neoplasm of the breast (C50), cervix uteri (C53), other and unspecified parts of the uterus (C54–C55), other female genital organs (C51–C52 and C56–C58), bladder (C67), and other malignant neoplasms of the urinary tract (C64–C66 and C68). The nervous system group included malignant neoplasms of the eye and adnexa (C69), brain (C71), other parts of the central nervous system (C70 and C72), and other, ill-defined, secondary, unspecified, and multiple sites (C73–C80 and C97). The lymphoid and hematopoietic system group included Hodgkin disease (C81), non-Hodgkin lymphoma (C82–C86), leukemia (C91–C95), and other malignant neoplasms of the lymphoid, hematopoietic, and related tissues (C88–C90 and C96). The other group included malignant neoplasms of other, ill-defined, secondary, unspecified, and multiple sites (C73–C80 and C97).

### 2.4. Statistical Analysis

The crude person-years and percentages were calculated according to sex, age, and type of work. The age-standardized incidence ratios (SIRs) and 95% confidence interval (CI) of all cancer types in the aircraft transport industry workers compared to those in the government employee and the entire employee population were calculated. Age-standardization was conducted by 5-year standardization of age from 25 to 60 after stratification of gender at same time of cohort period. The 95% CI were estimated by mid-t tests. When both the SIR and the lower limit of 95% CI were greater than or equal to 1, we considered this to indicate a statistically significant increased risk of cancer in the aircraft transport industry group compared to the reference groups. All analyses were conducted using SAS, version 9.4 (SAS Institute, Cary, NC, USA). In Table 1, we described the study participants by age and all cancer cases according to the definition of cohort with gender stratification.

### 2.5. Ethical Considerations

The data used in this study were anonymized prior to its release to the authors from the National Health Insurance Service. The Institute Review Board (IRB) of the Yonsei University Health System approved the study design (IRB number: Y-2017-0100).

## 3. Results

Table 1 indicates a total 59,751 person-years in aircraft transport industry workers, 5,678,047 person-years in government employees, and 85,954,378 whole employee person-years included in the present study. There were higher percentages of male than female workers in all groups. Most of the younger workers were female.

**Figure 1:**
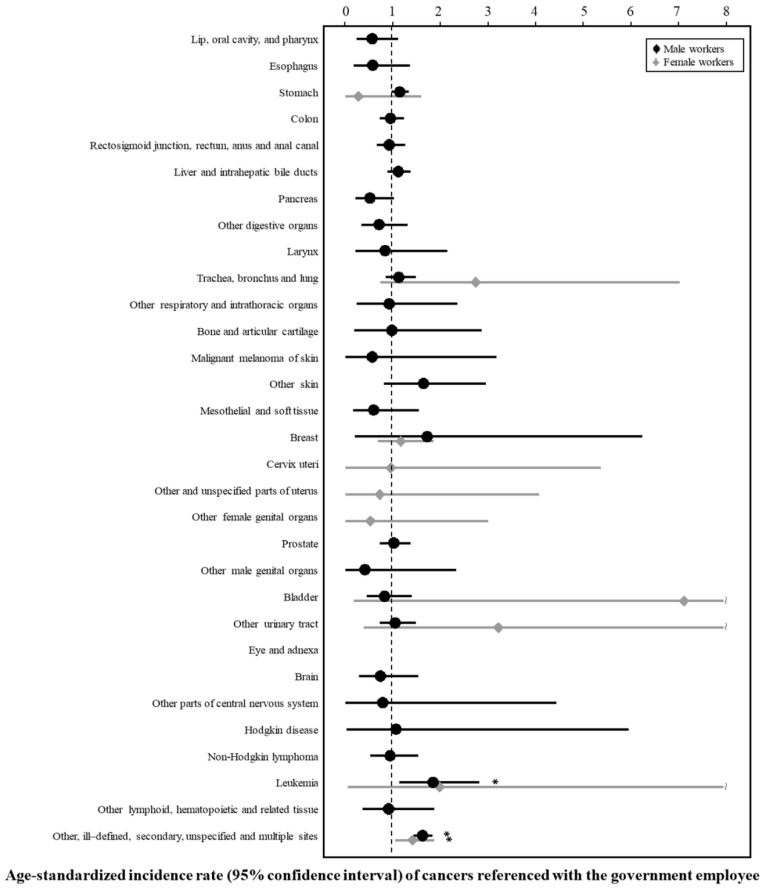
Age-standardized incidence rate (95% confidence interval) of cancers referenced with the government employee.

**Figure 2:**
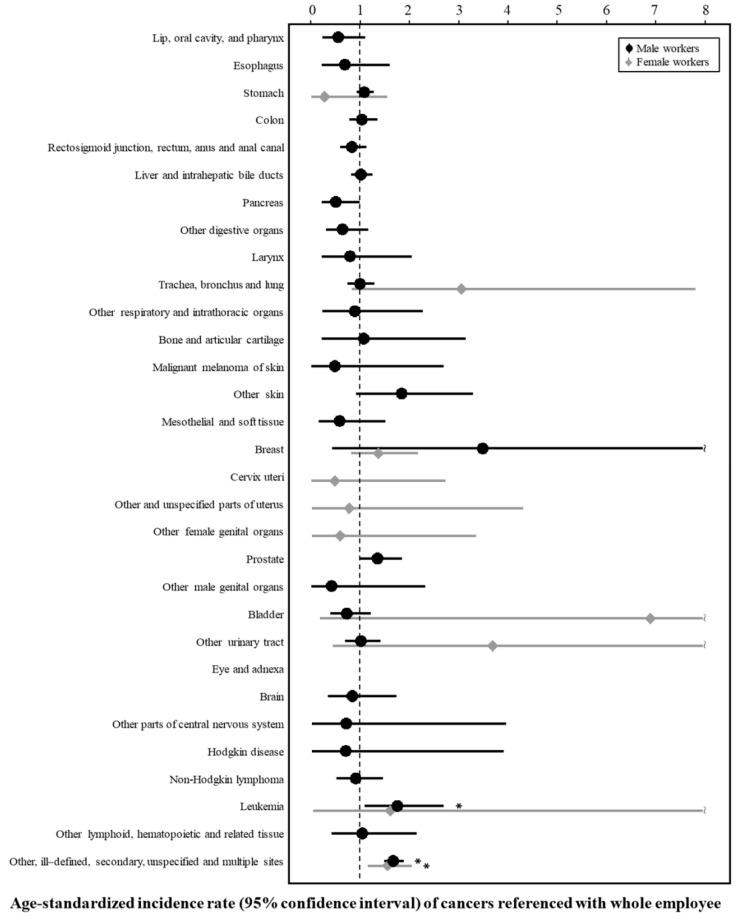
Age-standardized incidence rate (95% confidence interval) of cancers referenced with whole employee.

Table 2 and Figure 1 and Figure 2 present the SIRs and 95% CIs for cancers among male workers. The SIRs (95% CI) for leukemia (ICD-10; C91–C95) and malignant neoplasms of other, ill-defined, secondary, unspecified, and multiple sites (ICD-10; C73–C80, C97) were significantly higher for the aircraft workers than for the government employee group (1.86 (1.15–2.84) and 1.64 (1.45–1.85), respectively) and the whole employee group (1.77 (1.10–2.70) and 1.68 (1.49–1.89), respectively).

Table 3 and Figure 1 and Figure 2 show the SIRs and 95% CIs for cancers among female workers. The SIR could not be estimated for most cancers due to the lack of cases. The SIRs (95% CI) for malignant neoplasm of other, ill-defined, secondary, unspecified, and multiple sites (ICD-10; C73–C80, C97) were higher for the female workers in the aircraft transport industry group than for the government employee group (1.64 (1.45–1.85)) and the whole employee group (1.68 (1.49–1.89)).

## 4. Discussion

The aim of this study was to assess the risk of cancers in air transportation industry workers in Korea compared to other working populations. The study results showed statistically significant increased risks for incident leukemia in male air transportation industry workers compared to the reference groups after age standardization. Although female air transportation industry workers also had high incident leukemia ratios compared to the reference groups, these were not statistically significant. Both male and female workers had statistically significant higher ratios of other, ill-defined cancer incidence compared to their respective reference groups. Interestingly, the female workers had a statistically significant higher risk of incidence for all cancers compared to their reference groups, while the male workers had a statistically significant decrease for all cancers.

Through their occupation, air transportation industry workers, specifically flight attendants, are exposed to ionizing radiation of cosmic origin as well as other related health risk factors, including circadian dysrhythmia due to night shift work and long or irregular working hours during flights across multiple time zones, and poor cabin air quality from a number of sources [5]. Flight attendants constitute an occupational group with high radiation exposure. The annual radiation exposure dose in aircraft workers was 3.1 (range 0–9.5) mSv in Finnish aircraft cabin attendants [21] and 2.5 ± 1.0 in Pan American World Airways workers, which is in addition to the background radiation of the general population [22]. These exposures are higher than the annual limit of 1 mSv recommended by the International Commission for Radiological Protection (ICRP) [23]. Although the cumulative occupational lifetime dose of ionizing radiation generally remains below 100 mSv [24], there is uncertainty in the estimation of the cumulative dose for individual flight attendants due to several reasons including the lack of individual flight histories. Furthermore, cosmic rays can fluctuate due to solar activity, with the amount of exposed energy increasing by several thousand percent in high solar activity events [25]. During a solar storm, a person flying over the North Pole could receive almost an entire year’s worth of radiation exposure in just one flight segment [26].

These chronic and unpredictable radiation exposures may be associated with carcinogenicity in air transportation workers. Cosmic radiation as a form of ionizing radiation can damage DNA in living cells and lead to chromosomal aberrations that may result in neoplastic transformation [27]. Leukemia is a well-known neoplastic disorder related to ionizing radiation. Many studies have reported a higher incidence of leukemia among flight attendants, although the results were inconclusive due to the relatively small number of cases. Increased deletion or loss of chromosome 7 has been observed in patients with myelodysplasia and acute myeloid leukemia (AML) in cohorts comprising of aircrews [28]. The excess odds ratio [OR] of 1.66 per 10 mSv for non-chronic lymphocytic leukemia in a cohort study of four Nordic countries, based on nine cases, was not statistically significant, while the SIR for AML was 1.83 based on six observed cases and thus non-significant. A Danish population-based cohort study found evidence suggestive of an increased risk of AML with increasing flight hours in commercial jet cockpit crews [29].

The results regarding individual cancer sites must be interpreted with caution since the number of incident cases was small, as in previous studies, with statistically non-significant findings. As researchers have become aware of this, they have attempted to conduct pooled analyses and meta-analyses; however, the results of such analyses remain inconclusive. Moreover, a healthy worker effect is clearly apparent.

Generally, healthy workers are enrolled as pilots and flight attendants who are prepared to handle customer emergencies. By necessity of their profession, flight attendants have strict requirements regarding their fitness for the job and these occupational groups are subjected to extensive and continuous selection. Most findings to date have reported a relatively low overall mortality rate of cockpit crews, indicating a strong healthy worker effect, as well as reflecting crew members’ high socioeconomic status [27,30,31]. These crews also undergo strict medical surveillance, leading to even greater health advantages. However, early detection of cancer may result in higher rates of cancer incidence at lower stages of tumor development that would not increase mortality rates [12]. This may be a source of detection bias, particularly when comparing flight attendants to the general population. In the current study, risk of all cause cancer morbidity was about two times higher in female air transportation industry workers compared to the whole working population, while the risk of all cancer morbidity in males was about 40% less. This difference may be due to hazard exposure pattern and dose, biological response to exposure, socioeconomic status between the two groups, or stronger healthy worker effect and stricter requirements in cockpit crews. Because the assessment of morbidity has limitation for estimating actual cancer risk, especially among groups who are heathy but had earlier diagnoses, further analysis assessing mortality rate is warranted.

There are several strengths of our study. First, the use of incident cancers as outcome events instead of cancer deaths is a clear advantage because the study power could be increased due to the larger number of cases. It also allowed for the evaluation of risks for cancers that are less fatal, such as skin cancer. Second, the study is also strengthened by the large sample size based on NHIS data for individuals with a long follow-up period with respect to the entire Korean population of insured employees.

However, there were some notable limitations. First, the lack of data on exposure among air transportation industry workers limited the power of the present study to identify the specific causes of cancer. Our study was also limited by the lack of information on lifestyle factors such as smoking, alcohol and drug use, leisure time sun exposure, and dietary factors which might have influenced the development of malignant diseases. Another important limitation was that there are many different professional positions in the air transportation industry, including cabin crew, cockpit crew, air transportation control officers, aircraft maintenance crew, and ground staff. Therefore, air transportation industry workers are exposed to different risk factors depending on their professional position. By aggregating these different positions into a single “exposed” group, the potential risks related to the working conditions in the air transportation sector are likely to have been underestimated. Because industrial type also represents other characteristics such as socio-economic status including education, there might have been an under or over-estimation problem [32,33]. In addition, we used relatively more strict definitions of air transportation than both reference groups, which may have also resulted in an underestimated effect size. Although there was an underestimation of cancer risks, some cancers still showed significantly increased risks.

To the best of our knowledge, this is the first study to investigate the risks of cancer among air transportation industry workers in Korea. Our nationally representative cohort highlighted the high risk of leukemia among air transportation industry workers. We hope that these findings will guide future research regarding the health of air transportation industry workers, a topic that has been understudied thus far. In particular, studies clarifying the risk of cancers in association with cosmic radiation are warranted. These should evaluate individual flight histories (flight hours, duration of employment, routes flown, and levels of exposure to cosmic radiation), lifestyle factors, and cancer characteristics (for example, subtypes of leukemia, tumor latency, and diagnosis by time periods). More detailed characterization of these risk factors will permit a better analysis of the situation. The European Union (EU) requires airlines to monitor radiation dose, organize schedules to reduce radiation exposure, and conduct cohort studies. Considering that air transportation industry workers in Korea are subject to fewer protections relative to flight attendants working in the EU, it is essential to urgently conduct high-quality studies.

## Figures and Tables

**Table 1:** The characteristics of the study participants at the baseline of cohort.

	Person-year, %					
	Air Transportation Industry Workers		Government Employee		Whole Employee	
Total person-years	59,751		5,678,047		85,954,378	
Gender						
Male	45,673	76.4	2,898,213	51.0	57,871,482	67.3
Female	14,078	23.6	2,779,834	49.0	28,082,896	32.7
Age						
Total						
25–30	5980	10.0	290,037	5.1	9,240,276	10.8
31–35	11,398	19.1	722,694	12.7	15,509,508	18.0
36–40	12,109	20.3	974,979	17.2	16,435,257	19.1
41–45	10,330	17.3	1,054,586	18.6	14,852,909	17.3
46–50	8719	14.6	1,056,016	18.6	12,825,823	14.9
51–55	7108	11.9	920,516	16.2	10,270,762	11.9
56–60	4107	6.9	659,219	11.6	6,819,843	7.9
Male						
25–30	2378	5.2	57,160	2.0	4,240,331	7.3
31–35	6997	15.3	210,875	7.3	9,019,025	15.6
36–40	9883	21.6	390,711	13.5	11,470,377	19.8
41–45	9102	19.9	516,571	17.8	10,897,150	18.8
46–50	7615	16.7	615,957	21.3	9,434,555	16.3
51–55	6121	13.4	622,025	21.5	7,639,526	13.2
56–60	3577	7.8	484,914	16.7	5,170,518	8.9
Female						
25–30	3602	25.6	232,877	8.4	4,999,945	17.8
31–35	4401	31.3	511,819	18.4	6,490,483	23.1
36–40	2226	15.8	584,268	21.0	4,964,880	17.7
41–45	1228	8.7	538,015	19.4	3,955,759	14.1
46–50	1104	7.8	440,059	15.8	3,391,268	12.1
51–55	987	7.0	298,491	10.7	2,631,236	9.4
56–60	530	3.8	174,305	6.3	1,649,325	5.9

**Table 2:** The age-standardized incidence ratios (SIR) and 95% confidence interval (CI) of the air transportation industry male workers for cancers according to the reference group.

Cancer	ICD-10	Reference, SIR (95% CI) for Cancers	
		Government Employee	Whole Employee
All cancer		0.58 (0.54–0.62)	0.57 (0.53–0.61)
Gastrointestinal tract system			
Malignant neoplasms of lip, oral cavity, and pharynx	C00–C14	0.58 (0.25–1.13)	0.56 (0.24–1.11)
Malignant neoplasm of esophagus	C15	0.59 (0.19–1.37)	0.69 (0.22–1.61)
Malignant neoplasm of stomach	C16	1.16 (0.98–1.35)	1.09 (0.93–1.28)
Malignant neoplasm of colon	C18	0.96 (0.73–1.25)	1.04 (0.79–1.36)
Malignant neoplasms of rectosigmoid junction, rectum, anus, and anal canal	C19–C21	0.93 (0.67–1.27)	0.84 (0.60–1.14)
Malignant neoplasm of liver and intrahepatic bile ducts	C22	1.13 (0.90–1.39)	1.02 (0.82–1.26)
Malignant neoplasm of pancreas	C25	0.52 (0.23–1.04)	0.51 (0.22–1.00)
Other malignant neoplasms of digestive organs	C17, C23–C24, C26	0.72 (0.34–1.32)	0.64 (0.31–1.17)
Respiratory system			
Malignant neoplasm of larynx	C32	0.85 (0.23–2.16)	0.80 (0.22–2.06)
Malignant neoplasm of trachea, bronchus, and lung	C33–C34	1.14 (0.86–1.49)	1.00 (0.75–1.30)
Other malignant neoplasms of respiratory and intrathoracic organs	C30–C31, C37–C39	0.93 (0.25–2.37)	0.90 (0.24–2.29)
Bone and skin			
Malignant neoplasms ofbone and articular cartilage	C40–C41	0.99 (0.20–2.89)	1.08 (0.22–3.16)
Malignant melanoma of skin	C43	0.58 (0.01–3.21)	0.49 (0.01–2.71)
Other malignant neoplasm of skin	C44	1.66 (0.83–2.97)	1.85 (0.92–3.31)
Malignant neoplasms of mesothelial and soft tissue	C45–C49	0.61 (0.17–1.56)	0.59 (0.16–1.52)
Genitourinary tract system			
Malignant neoplasm of prostate	C61	1.03 (0.74–1.39)	1.36 (0.98–1.85)
Other malignant neoplasms of male genital organs	C60, C62–C63	0.42 (0.01–2.35)	0.42 (0.01–2.34)
Malignant neoplasm of bladder	C67	0.84 (0.46–1.42)	0.73 (0.40–1.22)
Other malignant neoplasms of urinary tract	C64–C66, C68	1.06 (0.73–1.49)	1.02 (0.70–1.43)
Nervous system			
Malignant neoplasm of eye and adnexa	C69	None	None
Malignant neoplasm of brain	C71	0.75 (0.30–1.55)	0.85 (0.34–1.75)
Malignant neoplasm of other parts of central nervous system	C70, C72	0.80 (0.02–4.46)	0.72 (0.02–3.98)
Lymphoid and hematopoietic system			
Hodgkin disease	C81	1.08 (0.03–5.99)	0.71 (0.02–3.93)
Non-Hodgkin lymphoma	C82–C86	0.95 (0.54–1.54)	0.91 (0.52–1.47)
Leukemia	C91–C95	1.86 (1.15–2.84)	1.77 (1.10–2.70)
Other malignant neoplasms of lymphoid, hematopoietic and related tissue	C88–C90, C96	0.92 (0.37–1.89)	1.05 (0.42–2.16)
Other			
Malignant neoplasm of other, ill-defined, secondary, unspecified, and multiple sites	C73–C80, C97	1.64 (1.45–1.85)	1.68 (1.49–1.89)

**Table 3:** The age-standardized Incidence Ratios (SIR) and 95% confidence interval (CI) of the air transportation industry female workers for cancers according to reference group.

Cancer	ICD-10	Reference, SIR (95% CI) for Cancers	
		Government Employee	Whole Employee
All cancer		2.27 (1.79–2.84)	2.09 (1.65–2.62)
Gastrointestinal tract system			
Malignant neoplasms of lip, oral cavity, and pharynx	C00–C14	None	None
Malignant neoplasm of esophagus	C15	None	None
Malignant neoplasm of stomach	C16	0.29 (0.01–1.61)	0.28 (0.01–1.55)
Malignant neoplasm of colon	C18	None	None
Malignant neoplasm of rectosigmoid junction, rectum, anus, and anal canal	C19–C21	None	None
Malignant neoplasm of liver and intrahepatic bile ducts	C22	None	None
Malignant neoplasm of pancreas	C25	None	None
Other malignant neoplasm of digestive organs	C17, C23–C24, C26	None	None
Respiratory system			
Malignant neoplasm of larynx	C32	None	None
Malignant neoplasm of trachea, bronchus, and lung	C33–C34	2.76 (0.76–7.06)	3.07 (0.84–7.85)
Other malignant neoplasms of respiratory and intrathoracic organs	C30–C31, C37–C39	None	None
Bone and skin			
Malignant neoplasms ofbone and articular cartilage	C40–C41	None	None
Malignant melanoma of skin	C43	None	None
Other malignant neoplasm of skin	C44	None	None
Malignant neoplasms of mesothelial and soft tissue	C45–C49	None	None
Genitourinary tract system			
Malignant neoplasm of breast	C50	1.18 (0.70–1.87)	1.38 (0.82–2.18)
Malignant neoplasm of cervixuteri	C53	0.97 (0.02–5.40)	0.49 (0.01–2.74)
Malignant neoplasms of other and unspecified parts of uterus	C54–C55	0.74 (0.02–4.11)	0.78 (0.02–4.33)
Other malignant neoplasms of female genital organs	C51–C52, C56–C58	0.54 (0.01–3.02)	0.60 (0.02–3.37)
Malignant neoplasm of bladder	C67	7.16 (0.18–39.90)	6.93 (0.8–38.61)
Other malignant neoplasms of urinary tract	C64–C66, C68	3.24 (0.39–11.72)	3.71 (0.45–13.40)
Nervous system			
Malignant neoplasm of eye and adnexa	C69	None	None
Malignant neoplasm of brain	C71	None	None
Malignant neoplasm of other parts of central nervous system	C70, C72	None	None
Lymphoid and hematopoietic system			
Hodgkin disease	C81	None	None
Non-Hodgkin lymphoma	C82–C86	None	None
Leukemia	C91–C95	2.00 (0.05–11.16)	1.62 (0.04–9.00)
Other malignant neoplasms of lymphoid, hematopoietic and related tissue	C88–C90, C96	None	None
Other			
Malignant neoplasm of other, ill-defined, secondary, unspecified, and multiple sites	C73–C80, C97	1.43 (1.06–1.89)	1.57 (1.16–2.06)

## Approval of the Research Protocol

The data in this study were anonymized prior to its release to the authors from the National Health Insurance Service. The Institute Review Board (IRB) of the Yonsei University Health System approved the study design (IRB number: Y-2017-0100).
